# Systematic review on the value of end-of-treatment FDG-PET in improving overall survival of lymphoma patients

**DOI:** 10.1007/s00277-019-03881-x

**Published:** 2019-12-07

**Authors:** Hugo J.A. Adams, Thomas C. Kwee

**Affiliations:** 1 Department of Radiology and Nuclear Medicine, Academic Medical Center, University of Amsterdam, Meibergdreef 9, 1105AZ, Amsterdam, Netherlands; 2 Department of Radiology and Nuclear Medicine, University Medical Center Groningen, University of Groningen, Groningen, Netherlands

**Keywords:** End-of-treatment, FDG-PET, Lymphoma, Response assessment, Overall survival, Therapy

## Abstract

This study aimed to systematically review the value of end-of-treatment ^18^F-fluoro-2-deoxy-D-glucose (FDG) positron emission tomography (PET) in improving overall survival (OS) of lymphoma patients. Medline was systematically searched for (1) randomized trials comparing the OS of patients who underwent end-of-treatment FDG-PET to those without and FDG-PET-based end-of-treatment evaluation and for (2) non-randomized studies comparing the OS of patients who underwent end-of-treatment FDG-PET to a (historical) cohort of patients without an FDG-PET-based end-of-treatment evaluation. The Medline search revealed 6284 articles. However, none of these reported data on the value of end-of-treatment FDG-PET in improving OS of lymphoma patients. In conclusion, the present systematic review reveals that there is currently no study at all that evaluates the value of end-of-treatment FDG-PET in improving OS of lymphoma patients. As a result, it remains unknown whether end-of-treatment FDG-PET increases OS and in which lymphoma subtype these examinations are of particular value. Future studies are required to demonstrate its value in this setting before it can be recommended as an evidence-based diagnostic tool by guidelines on the use of imaging in lymphoma.

## Introduction

Lymphomas are the sixth most frequently occurring malignant tumors, comprise around 4.8% of cases of newly diagnosed cancer, and cause about 3.6% of all cancer-related deaths in the Western world [1]. Current international guidelines, such as the Lugano classification [2, 3], the guidelines of the European Society of Medical Oncology (ESMO) [4, 5], and the guidelines of the National Comprehensive Cancer Network [6, 7] propose an end-of treatment evaluation using ^18^F-fluoro-2-deoxy-D-glucose (FDG) positron emission tomography (PET) for lymphomas that are FDG-avid at baseline. These guidelines, however, are based on expert opinions, and the articles cited in these guidelines do not refer to studies showing patients undergoing an end-of-treatment FDG-PET evaluation to have a better survival than those who do not.

Over the past years, multiple studies [8] have been performed to determine whether an FDG-PET scan performed during treatment (interim FDG-PET) is useful in improving survival in lymphoma patients by applying therapy de-escalation in patients with an early FDG-PET-based response and applying therapy escalation in patients without an favourable FDG-PET-based response. However, it is unclear if there are any trials that analyzed whether an end-of-treatment FDG-PET evaluation results in a benefit for lymphoma patients in terms of an increase in overall survival (OS). An important difference between interim and end-of-treatment FDG-PET is that an end-of-treatment FDG-PET evaluation does not allow for treatment de-escalation, because the scan is acquired after the entire therapy regimen has already been applied. The main potential benefit of an end-of-treatment FDG-PET evaluation is that it may allow for an earlier identification of treatment failure to first-line therapy before symptomatic disease recurrence occurs. Theoretically, this may allow consolidation therapies to be applied at an earlier stage, and this may increase OS compared to an approach of watch-and-wait until the lymphoma becomes symptomatic. However, in order for this strategy to be effective, four needs have to be met. First, end-of-treatment FDG-PET needs to be a sensitive method to detect residual disease shortly after treatment. If not, early identification of patients who require consolidation therapy is not possible. Second, end-of-treatment FDG-PET needs to be specific in order to prevent large-scale implementation of unnecessary verification biopsies or erroneous initiation of consolidation treatments in case presumed residual disease is not histologically verified. Third, early (imaging directed) application of consolidation treatments must result in a benefit in terms of OS compared to an approach of watch-and-wait and initiation of consolidation therapies when the recurrent lymphoma becomes symptomatic. Fourth, the strategy of early imaging-based detection of therapy-resistant disease needs to be cost-effective.

In order to justify an end-of-treatment FDG-PET evaluation, a clear benefit in terms of OS needs to be proven over the approach of watch-and-wait until symptoms occur. Therefore, the present systematic review aimed to assess whether a policy of routine application of end-of-treatment FDG-PET results in a benefit in terms of OS compared to an approach of watch-and-wait until development of symptoms.

## Materials and methods

### Search strategy

The Medline database was searched for original studies comparing the OS of patients undergoing end-of-treatment FDG-PET to those who did not undergo end-of-treatment FDG-PET. The search was performed on August 10, 2019, and is displayed in Table 1. Bibliographies of included studies were scrutinized for appropriate studies not retrieved by the Medline search. Titles and abstracts were screened using Thomson Reuters Endnote 7 for Mac.

**Table 1:** Search strategy and results as on August 10, 2019

No.	Search string	No. of hits in Medline
1.	Lymphoma OR lymphomas OR Hodgkin OR Hodgkin’s	270,175
2.	Fluorodeoxyglucose OR 2-fluoro-2-deoxy-D-glucose OR FDG OR positron emission tomography OR PET OR FDG-PET	127,586
3.	No. 1 AND No. 2	6284

### Study selection

Randomized or non-randomized original studies reporting the number of deaths or OS of patients undergoing end-of-treatment FDG-PET (using either a stand-alone PET or hybrid PET/CT system) compared to the number of deaths/OS of those without end-of-treatment FDG-PET evaluation were eligible for inclusion. In addition, both prospective and retrospective studies comparing the outcome of patients with an end-of-treatment FDG-PET evaluation to a (historical) cohort of patients without an end-of-treatment FDG-PET evaluation (pre-post study design) were regarded suitable. Studies including patients with either Hodgkin’s lymphoma, aggressive non-Hodgkin’s lymphoma, or indolent non-Hodgkin’s lymphoma were considered. Studies were suitable if a comparison of OS of patients with an end-of-treatment FDG-PET evaluation was made with a watch-and-wait policy until symptomatic disease occurred or any other kind of end-of-treatment evaluation including computed tomography (CT), laboratory tests, or clinical evaluation. Studies without original patient data (e.g., guidelines, letters, editorials, reviews, and meta-analyses) and conference abstracts were excluded. Studies were only included when written in English, French, Spanish, German, Italian, or Dutch. Studies including less than 10 patients were excluded. Eligible articles written by the same authors or performed at the same institution were screened for duplication of data reporting. In case of duplicate data reporting, the article reporting the largest number of patients was included. Initially, titles and abstract retrieved by the Medline search were screened for eligibility. At a later stage, the full-text versions of the remaining articles were explored in detail using the aforementioned in- and exclusion criteria.

### Statistical analysis

The odds ratio with corresponding 95% confidence interval (95% CI) for the number of events of deaths during follow-up for patients with end-of-treatment FDG-PET versus those without was calculated for each individual study. If there was heterogeneity among individual studies (defined as I^2^ ≥ 50%), a random effects model was used to calculate the pooled odds ratio, whereas in absence of heterogeneity (defined as defined as I^2^ < 50%), a fixed effects model was used. For studies that did not report the number of events during follow-up but only the OS (which does not allow for data pooling), the results were descriptively analyzed.

## Results

### Literature search

The Medline search revealed 6284 articles. Of these, 183 were potentially eligible and read in full-text format. All of these 183 articles were excluded, all because these studies did not report a comparison of OS of patients with end-of-treatment FDG-PET evaluations with those of a cohort without end-of-treatment FDG-PET evaluations. Therefore, 0 studies remained.

### Randomized trials evaluating the outcome of lymphoma patients with end-of-treatment FDG-PET evaluation versus those without

The systematic search did not reveal any randomized trials evaluating the effect of an end-of-treatment FDG-PET evaluation on OS of lymphoma patients.

### Studies comparing the outcome of patients with an end-of-treatment FDG-PET evaluation to a (historical) cohort without an end-of-treatment FDG-PET evaluation (pre-post study design)

The systematic search did not reveal any studies comparing the OS of patients with an end-of-treatment FDG-PET evaluation to a (historical) cohort of patients without an end-of-treatment FDG-PET evaluation.

## Discussion

The present systematic review revealed that, at present, there is no randomized controlled trial available evaluating whether performing end-of-treatment FDG-PET imaging in lymphoma patients results in a better patient outcome than end-of-treatment evaluations by means of CT, laboratory values, or performing no routine end-of-treatment evaluation at all. Furthermore, there is no study that compares the outcome of patients who underwent end-of-treatment FDG-PET imaging with a historical cohort of patients at a time when routinely performing FDG-PET after treatment was not implemented yet in standard patient care (pre-post design). Consequently, it remains unknown whether end-of-treatment FDG-PET imaging really benefits lymphoma patients. This finding is of major importance, because international guidelines such as the Lugano classification [2, 3], the guidelines of the European Society of Medical Oncology (ESMO) [4, 5], and the guidelines of the National Comprehensive Cancer Network [6, 7] propose an end-of treatment evaluation using FDG-PET for all lymphomas that are FDG-avid at baseline. Consequently, end-of-treatment FDG-PET evaluations are implemented in routine care, and annually thousands of patients undergo an end-of-treatment FDG-PET evaluation worldwide, although a scientific base for this practice is completely lacking.

The lack of studies on the value of end-of-treatment FDG-PET in improving patient outcome highly contrasts to the extensive research that has been performed on the value of interim FDG-PET scans [8] that are performed during anti-lymphoma treatment. Over the last years, numerous observational studies evaluating the prognostic value of interim FDG-PET results, as well as experimental studies in which treatment is adapted on basis of the interim FDG-PET result, have been published. These studies applied treatment de-escalation in patients if FDG-PET suggested a favorable response after a few cycles of (immuno)chemotherapy, whereas treatment intensification was applied if FDG-PET showed a less favorable response. However, a major difference between interim and end-of-treatment FDG-PET is that end-of-treatment FDG-PET does not allow therapy de-escalation because scans are acquired after the entire first-line regimen has been applied. Furthermore, end-of-treatment FDG-PET does not allow for early treatment intensification in patients who cannot be cured by first-line therapies. The only benefit end-of-treatment FDG-PET can offer in terms of improving patient outcome is that early detection of residual disease by means of imaging, before the patient becomes asymptomatic, may result in earlier application of additional radiation therapy or second-line (immuno)chemotherapy. However, whether this approach will truly result in an improved outcome for the lymphoma patients remains unknown, as indicated by the present systematic review. On the other hand, numerous previous studies have shown that early detection of disease recurrence in patients who achieved a complete remission by means of surveillance FDG-PET scans provides no survival benefit [9–15]. Although these results on surveillance FDG-PET imaging cannot be translated directly to the end-of-treatment setting, it demonstrated that early imaging-based detection of residual disease does not unambiguously translate into an OS improvement.

End-of-treatment FDG-PET evaluations in lymphoma patients may have advantages beyond improving outcome. For example, assessing disease status at an early stage may be used to inform the individual patient about his/her prognosis or may be used as outcome measure of therapeutic trials. However, several studies have shown that end-of-treatment does not always adequately reflect disease status. Due to the limited spatial resolution (6–7 mm) of current FDG-PET systems, end-of-treatment FDG-PET cannot detect small residual lymphoma deposits, which is underlined by the finding that actually a high number of patients in complete remission at end-of-treatment FDG-PET develop disease relapse during follow-up [16–18]. Of note, in baseline FDG-avid, indolent follicular lymphoma, a complete response does not equal disease eradication, because these lymphomas are generally regarded as incurable. On the other hand, several studies revealed high proportions of false-positive end-of-treatment FDG-PET results, with therapy-induced inflammation being responsible for the majority of these false-positive cases [19, 20]. Consequently, end-of-treatment FDG-PET may not be an adequate tool to inform the patient about his/her prognosis and may be invalid as an outcome measure for therapeutic trials. Another potential benefit of end-of-treatment FDG-PET is that early detection of residual lymphomatous deposits at a stage in which the disease is less extensive than would be in case of watchful waiting may enable the application of radiation therapy rather than systematic second-line (immuno)chemotherapy, which is generally much more toxic.

On the other hand, it should be realized that systematically performing end-of-treatment FDG-PET scans in all lymphoma patients with FDG-avid lymphoma comes at a price. First, FDG-PET scans are costly and may not be available in second- and third-world countries. Second, the application of FDG-PET imaging is associated with a non-negligible exposure to ionizing radiation and may cause patient discomfort (both the preparation and the acquisition of the PET images). Third, false-positive results may cause unnecessary anxiety to the patient. False-positive lesions at end-of-treatment FDG-PET may also drive the treating physician to perform (superfluous) biopsies with associated costs and risks. In addition, when it is not possible to perform a biopsy, false-positive end-of-treatment FDG-PET results may lead to erroneous initiation of futile additional radiotherapy or even highly toxic second-line (immuno)chemotherapies.

This systematic review had one major drawback, namely, that not any study was included due to a complete lack of evidence on this topic. Consequently, it remains unknown whether an FDG-PET-based treatment evaluation benefits lymphoma patients as compared to other (cheaper and/or more patient friendly) approaches such as clinical examination, laboratory assessments, CT, or by not performing any end-of-treatment evaluation at all. On the other hand, this finding underlines the importance of the present systematic review, because end-of-treatment FDG-PET imaging is proposed by international guidelines and is already implemented in routine clinical care. Due to the lack of any trial on this topic, it cannot be determined whether systematically performing end-of-treatment FDG-PET evaluations are effective in improving outcome and whether this approach is cost-effective, in which lymphoma subtype or in which setting the benefit of end-of-treatment FDG-PET is sufficiently high. Theoretically, the value of an end-of-treatment FDG-PET evaluation may be lower after first-line treatment of early-stage Hodgkin’s lymphoma and favorable aggressive non-Hodgkin’s lymphoma, because in these settings, the probability of treatment failure is relatively low. Consequently, the probability of detecting treatment failure at an early stage, and subsequent early initiation of second-line treatments, is also low. In indolent (follicular) lymphoma, the results of end-of-treatment FDG-PET evaluations may also be of limited value, because these lymphomas are generally considered incurable. Furthermore, in patients with indolent lymphoma and a positive end-of-treatment FDG-PET result, there is rarely an indication to directly apply second-line treatment, since these patients are treated only when symptomatic disease develops. Therefore, end-of-treatment is FDG-PET which is unlikely to prolong OS of follicular lymphoma patients. End-of-treatment FDG-PET may theoretically be of greatest value in high-risk aggressive lymphomas or after second, third, or later lines of therapy, in which the probability of treatment failure is relatively high.

In conclusion, the present systematic review reveals that there is currently no study at all that evaluates the value of end-of-treatment FDG-PET in improving OS of lymphoma patients. As a result, it remains unknown whether end-of-treatment FDG-PET increases OS and in which lymphoma subtype these examinations are of particular value. Future studies are required to demonstrate its value in this setting before it can be recommended as an evidence-based diagnostic tool by guidelines on the use of imaging in lymphoma.
